# Supercritical Fluid Extraction with CO_2_ of *Curcuma longa* L. in Comparison to Conventional Solvent Extraction

**DOI:** 10.3390/pharmaceutics14091943

**Published:** 2022-09-14

**Authors:** Ann-Kathrin Widmann, Martin A. Wahl, Dietmar R. Kammerer, Rolf Daniels

**Affiliations:** 1 Department of Pharmaceutical Technology, Eberhard Karls University, Auf der Morgenstelle 8, 72076 Tuebingen, Germany; 2 Department of Analytical Development and Research, Section Phytochemical Research, WALA Heilmittel GmbH, Dorfstrasse 1, 73087 Bad Boll, Germany

**Keywords:** supercritical fluid extraction, turmeric, carbon dioxide, stability study, HPLC, LC-MS, pressure, temperature

## Abstract

*Curcuma longa* L. is a traditional medicinal and spice plant containing a variety of lipophilic active substances with promising therapeutic properties. In this work, the solvent properties of supercritical carbon dioxide in a pressure and temperature range of 75–425 bar and 35–75 °C were investigated when *Curcuma longa* rhizomes were extracted. The three main curcuminoids, namely curcumin, demethoxycurcumin, and bisdemethoxycurcumin, together with the three main constituents of the essential oil, i.e., ar-turmerone, α-turmerone, and β-turmerone, were analyzed in the resulting extracts. For statistical evaluation, experiments were performed employing a full factorial design, in which flow rate, extraction time, and drug load were kept constant. Within the given conditions, the experimental design revealed an optimum yield of all aforementioned substances, when supercritical carbon dioxide extraction was performed at 425 bar and 75 °C. For comparison, solvent extracts using methanol and *n*-hexane were prepared and their main components were characterized using LC-MS. The stability of the extracts was monitored upon storage for 6 months at 22 and 40 °C under protection from light. The decomposition of individual compounds was mainly observed in the presence of residual water in the extracts.

## 1. Introduction

Turmeric (Curcumae longae rhizoma) is a traditional medicinal plant containing lipophilic active substances with promising therapeutic properties. These constituents belong to the curcuminoids with their main representative curcumin and contain essential oil which predominantly includes turmerones [1]. Curcuminoids are often recovered by conventional solvent extraction [2]. They are known for their intense yellow color and poor water solubility [3], as well as for their light sensitivity [4,5]. They are used for their antioxidant [6] and anti-inflammatory [7] properties as well as their anticancerogenic [8], antimutagenic [9], and antifungal [10] effects. The essential oil has been demonstrated to have antimicrobial [11], in vitro anticancer [12], in vivo antifungal [13], in mice antivenom [14], and insect repellent [15] properties. Turmeric oils or oleoresins are conventionally produced with hydro- or steam distillation [16], Soxhlet extraction [17], or microwave-assisted extraction [18], but can be obtained using supercritical extraction with CO_2_ as well [19].

Supercritical CO_2_ (scCO_2_) extraction is an elegant alternative to lipophilic solvent extraction, as it avoids the often laborious and expensive removal of potentially toxic solvent residues. Due to its significantly lower dynamic viscosity and only insignificantly lower density compared to liquid solvents, scCO_2_ provides favorable mass transport behavior [20,21,22]. Moreover, solubility of the target compounds can be modulated by influencing the density which can be adjusted by simply varying pressure and temperature [23,24,25]. The density can be increased by increasing the pressure or lowering the temperature. In addition, solubility can be modified towards more polar substances with the use of co-solvents such as organic solvents (e.g., methanol, ethanol) or water, however, the removal of the used co-solvents from the resulting extract can be challenging [26]. ScCO_2_ is a nondipolar solvent, and due to its low dielectric constant, it was historically treated as a nonpolar solvent [22,27] which turned out to be an oversimplification of its properties. More recent research shows that CO_2_ still is a polar molecule and, therefore, it has been described as a quadrupolar solvent that can participate in Lewis acid–base reactions [28]. 

The work presented here has been mainly focused on the change in solvent properties of pure scCO_2_ when evaluating curcumin, demethoxycurcumin (DMC), and bisdemethoxycurcumin (BDMC) as well as aromatic (ar-) turmerone, α-turmerone, and β-turmerone recovery over a defined pressure and temperature range of 75–425 bar and 35–75 °C, deliberately without the addition of any co-solvents. Moreover, the obtained scCO_2_ extracts were compared with conventional *n*-hexane and methanol extracts. Due to the different polarities of the solvents, different extraction patterns were expected representing the full range of extractables from turmeric. The aim of this study was to compare the quantitative composition of conventional methanol and hexane extracts with scCO_2_ extracts with respect to the aforementioned marker substances. Moreover, scCO_2_ extracts were studied for their stability upon storage for 6 months at 22 and 40 °C, respectively, under light protection.

## 2. Materials and Methods

### 2.1. Materials

Curcumae longae rhizomae pulv. subt. was purchased from Heinrich Klenk GmbH & Co. KG (Schwebheim, Germany). Carbon dioxide (Technical grade, ≥99.5%) was obtained from Westfalen AG (Muenster, Germany). Methanol (HPLC gradient grade) and *n*-hexane (HPLC grade) were purchased from Sigma-Aldrich Chemie GmbH (Taufkirchen, Germany). Formic acid (HPLC grade) was obtained from Carl Roth GmbH + Co. KG (Karlsruhe, Germany). Acetonitrile (HPLC grade) was purchased from Honeywell Riedel-de-Haën AG (Seelze, Germany). Highly purified water for HPLC was produced using a Purelab Option Q7, Veolia Water Technologies Deutschland GmbH (Celle, Germany). Potassium carbonate, Hydranal-Methanol dry, and Hydranal-Composite 5 were purchased from Honeywell International Inc. (Charlotte, NC, USA)). Acetonitrile (LC-MS grade) and formic acid (98%) for LC-MS analyses were obtained from Sigma-Aldrich (Steinheim, Germany). Analytical standards of curcumin and ar-turmerone were obtained from Sigma-Aldrich Chemie GmbH (Taufkirchen, Germany) and α-turmerone was obtained from Toronto Research Chemicals (Toronto, ON, Canada).

### 2.2. Solvent Extraction

Conventional solvent extraction was carried out as a two-step maceration, mixing 1 part of dried powdered turmeric with 2.5 parts of solvent (methanol and hexane, respectively; *m*/*m*). Maceration was carried out under exclusion of light by covering with aluminum foil and storing in a laboratory cupboard. After 24 h the suspension was filtered through a commercial cellulose paper filter, and 2.5 parts of the respective solvent were again added to the remaining insolubles. After a further 24 h both filtrates were combined and the solvent was removed with a rotary evaporator (Büchi, Labortechnik AG, Flawil, Switzerland).

### 2.3. Supercritical Fluid Extraction

**Figure 1:**
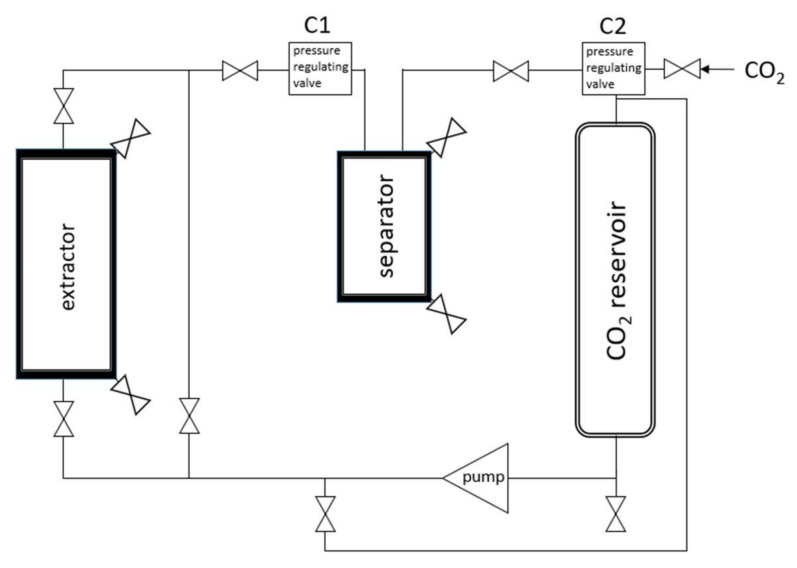
Simplified schematic depiction of the scCO_2_ pilot plant unit.

Supercritical fluid extraction was performed in a scCO_2_ Sietec-Sieber pilot plant unit (Sietec-Sieber, Maur, Switzerland) with dried powdered turmeric. Figure 1 illustrates a scheme of the scCO_2_ pilot plant unit. All experiments were performed using a constant drug load of 150 g for the design of experiments and 300 g for the extracts used in the stability study. The extraction was conducted at a flow rate of 5.0 kg/h for 1 h. Dissolved substances followed the stream of scCO_2_, which was pumped from the extractor into the separator. Extracted components were separated from scCO_2_ due to a sudden pressure relief behind the pressure-regulating valve C1 in the separator at 40 bar and 30 °C. This allowed the transition of scCO_2_ first into the liquid and subsequently into the gaseous state. This two-stage process prevented the extract from becoming a non-separable aerosol. The circular system allowed recycling of the CO_2_. The pressure and temperature of the extraction process were varied in a range of 75–425 bar and 35–75 °C, respectively, according to the design of the experiments (Table 1). To minimize differences due to the residual water contents of the dried powdered turmeric, the latter was preconditioned at 42% relative humidity for 7 days.

### 2.4. Liquid Chromatography with Mass Spectrometric Detection (LC-MS)

LC-MS measurements were carried out only for characterization of the main components measuring conventional solvent extracts in concentrations of 2–4 mg/mL dissolved in methanol. An Agilent 1200 HPLC (Agilent, Waldbronn, Germany) system equipped with a vacuum degasser (G1379B), a binary pump (G1312A), an autosampler (G1329A), a thermostatic column compartment (G1316A), and a diode array detector (G1315B) was used for chromatographic separation. A binary gradient eluent system (mobile phases A: 0.1% formic acid (*v*/*v*); B: acetonitrile) was applied using a SunFire C18 column 100 Å, 5 µm, 4.6 mm × 250 mm (Waters GmbH, Eschborn, Germany) in combination with a Nucleosil 100-5 EC 4/3 precolumn (Macherey-Nagel GmbH and Co. KG, Dueren, Germany) at a flow rate of 1.0 mL/min with the following gradient: 0–11 min, 35% B; 11–25 min, 35–70% B; 25–35 min, 70–100% B; 35–40 min, 100% B; 40–45 min, 100–35% B. The column oven was set at 25 °C and the injection volume was set at 20 µL. For detection, the wavelengths were set at 250, 265, and 425 nm. Mass spectrometric analyses were performed with an HCT ultra ion trap MS detector with an APCI ion source (Bruker Daltonik, Bremen, Germany). The device parameters were applied as follows: dry gas flow rate (N_2_), 4 L/min; nebulizer pressure, 50 psi; and capillary temperature, 325 °C; MS spectra were generated in the positive ionization mode with a compound stability and trap drive level of 100% in a range of *m*/*z* 50–1500. For data acquisition and processing the software Agilent Chemstation (Rev. B.01.03 SR1) (Agilent, Waldbronn, Germany) and Bruker Daltonik Esquire Control (version 6.1) (Bruker Daltonik GmbH, Bremen, Germany) were used.

### 2.5. High Performance Liquid Chromatography with Diode Array Detection (HPLC-DAD)

Quantification of all extracts included in the experimental design and the stability study was carried out with HPLC-DAD. All samples were assayed with HPLC using a Shimadzu HPLC system (Shimadzu Europa GmbH, Duisburg, Germany) equipped with a degassing unit (DGU-405), a solvent delivery module (LC-40D), an autosampler (SIL-40C), a column oven (CTO-40S), and a photo diode array detector (SPC-M49). A binary gradient eluent system (mobile phases A: 0.1% formic acid (*v*/*v*); B acetonitrile) was applied using a SunFire C18 column 100 Å, 5 µm, 4.6 mm × 250 mm (Waters GmbH, Eschborn, Germany) in combination with a Nucleosil 100-5 C8CC/3 precolumn (Macherey-Nagel GmbH and Co. KG, Dueren, Germany) at a flow rate of 1.0 mL/min with the following gradient: 0–20 min, 40–75% B; 20–25 min, 75–80% B; 25–28 min, 80–90% B; 28–31 min, 90% B; 31–32 min, 90–40% B; 32–36 min; 40% B. The column oven was set at 30 °C and the injection volume was set at 10 µL. The standard curcumin was detected at 425 nm with a retention time of 13.6 min, ar-turmerone at 250 nm after 23.8 min, and α-turmerone at 250 nm after 27.5 min. Curcumin was used for quantification of all three curcuminoids and ar-turmerone for the three regarded turmerones. Substances were quantified as curcumin or ar-turmerone equivalent using peak height values. To this end, equivalents to extract concentrations of 3.0–4.0 mg/mL for scCO_2_ extracts, 1.0–1.5 mg/mL for *n*-hexane extracts, and 0.3–0.5 mg/mL for methanol extracts were measured dissolved in methanol.

### 2.6. Design of Experiments (DoE)

For screening over the full scCO_2_ range that can be covered with the pilot plant unit used in the present study, a full factorial design was chosen, and the data were assessed using JMP 15.2 software (SAS Institute Inc., Cary, NC, USA) for experimental design and statistics. All experiments were performed in triplicate and 6 additional center points were included, which led to 33 experiments in total as specified in Table 1 The extracted substances were dissolved in methanol to allow complete withdrawal from the pilot plant unit. The methanolic solutions were assayed using HPLC-DAD. The extract yield was determined after the removal of methanol by rotary evaporation (Büchi, Labortechnik AG, Flawil, Switzerland).

### 2.7. Stability Study

For evaluating extract stability, only scCO_2_ extracts recovered under maximum pressure conditions were analyzed. To investigate the influence of dissolved CO_2_ during storage the extracts were withdrawn from the separator using a syringe without adding a solvent. For comparison, conventional solvent extracts recovered with methanol and *n*-hexane, respectively, and a scCO_2_ extract taken from the plant as a methanolic solution with subsequent removal of the solvent using a rotary evaporator (Büchi, Labortechnik AG, Flawil, Switzerland), were also examined as shown in Table 2. All extracts were prepared in triplicate and aliquots of 500 mg each were stored in 0.9 mL glass vials with a flare cap closure. Samples were stored at 22 and 40 °C, protected from light. Samples were taken 1, 28, 84, and 168 days after production for quantitating individual compounds using HPLC-DAD after dissolving the extracts in methanol.

### 2.8. Water Content Analysis after Karl Fischer

Water content was determined according to Ph. Eur 2.5.12 ‘water: semi-micro determination’ [29] based on the reaction of water with iodine (I_2_) and sulfur dioxide (SO_2_) as shown in following Equation (1).

2H_2_O + SO_2_ + I_2_ → H_2_SO_4_ + 2HI (1)

Simplified reaction principle of Karl Fischer’s titration of water with iodine (I_2_) and sulfur dioxide (SO_2_).

The analysis was performed in a 751 GPD Titrino (Deutsche METROHM GmbH & Co. KG, Filderstadt, Germany) and for the required anhydrous surrounding and solvent for the samples Hydranal-Methanol dry was used. The titrant applied was Hydranal-Composite 5, containing the necessary reagents iodine and sulfur dioxide as well as imidazole and 2-methylimidazol as basic components, all dissolved in diethylene glycol-monoethyl ether. In accordance with Ph. Eur., at first standardization was completed with pure water. Afterwards, suitability was tested with the scCO_2_ extract produced at 425 bar and 75 °C, taken from the plant as a methanolic solution with subsequent removal of the solvent and therefore considered to be water-free (Table 2, 42575R). The mean percentage recovery for the extract was 100.5%. For water determination, the regression line was calculated with the addition of water to the extract after Ph. Eur. and resulted in a slope of 1.004 and percentage errors of e_1_ = 0.4% and e_2_ = 0.0%. All samples were measured in duplicate.

## 3. Results and Discussion

### 3.1. Phytochemical Characterization of Solvent Extracts

Conventional turmeric methanol and hexane extracts were analyzed using LC-MS to characterize their main constituents. It was assumed that by covering both polar (methanol) and non-polar (*n*-hexane) extracts it should be possible to characterize all major compounds in either extract. Extracts recovered with scCO_2_ should yield phytochemical profiles in between those of the aforementioned two extremes.

**Figure 2:**
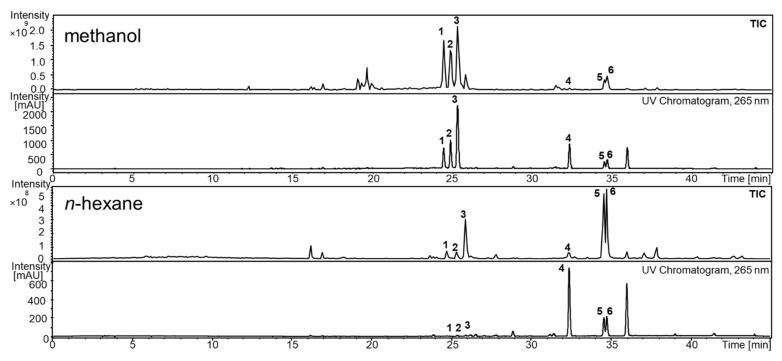
Total ion current (TIC) and UV chromatogram of methanol and *n*-hexane extracts; 1: bisdemethoxycurcumin (BDMC); 2: demethoxycurcumin (DMC); 3: curcumin; 4: ar-turmerone; 5: α-turmerone; and 6: β-turmerone.

Figure 2 shows the ultraviolet (UV) chromatograms and total ion currents (TIC) of the methanol and *n*-hexane extracts. Six major signals were number coded and based on their UV and MS data further characterized and assigned (Table 3). Curcumin, ar-turmerone, and α-turmerone were identified by comparison with the respective reference substances. BDMC, DMC, and β-turmerone were characterized using their MS spectra, which were compared with literature data.

### 3.2. Supercritical Carbon Dioxide Extraction

**Figure 3:**
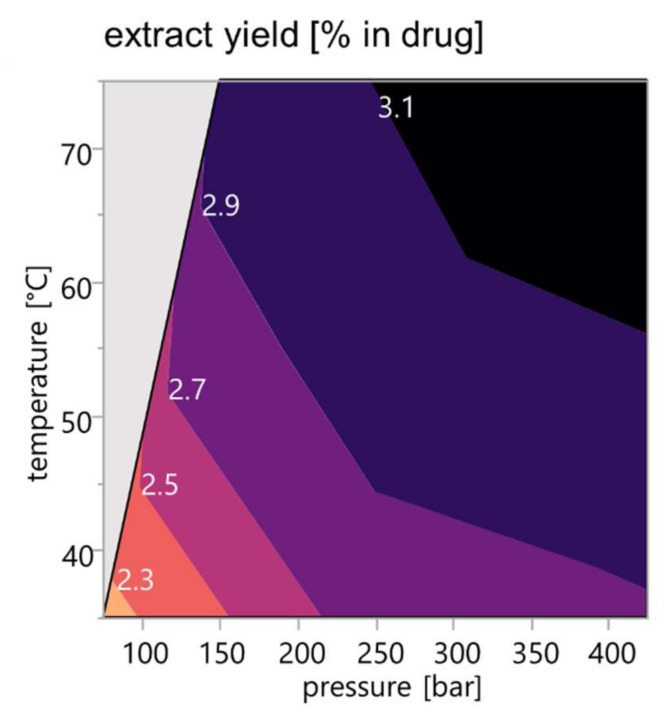
Response contour plot illustrating the extract yields depending on pressure and temperature; the area marked in grey represents combinations of pressure and temperature settings, which were not investigated; recovery rate related to the drug material in % (*m*/*m*).

To systematically study the influence of extraction conditions on the composition of turmeric scCO_2_ extracts, a full factorial experimental design was used. Figure 3 illustrates the extract yield recovered from the extractions of 150 g powdered drug load. It should be noted that the extract yields considered were obtained from methanolic solution and are therefore to be considered without co-extracted water. The yield of water-free extract ranges between below 2.3% for extraction settings under 100 bar and 40 °C and above 3.1% for pressure settings above 300 bar in combination with high-temperature settings above 60 °C.

**Figure 4:**
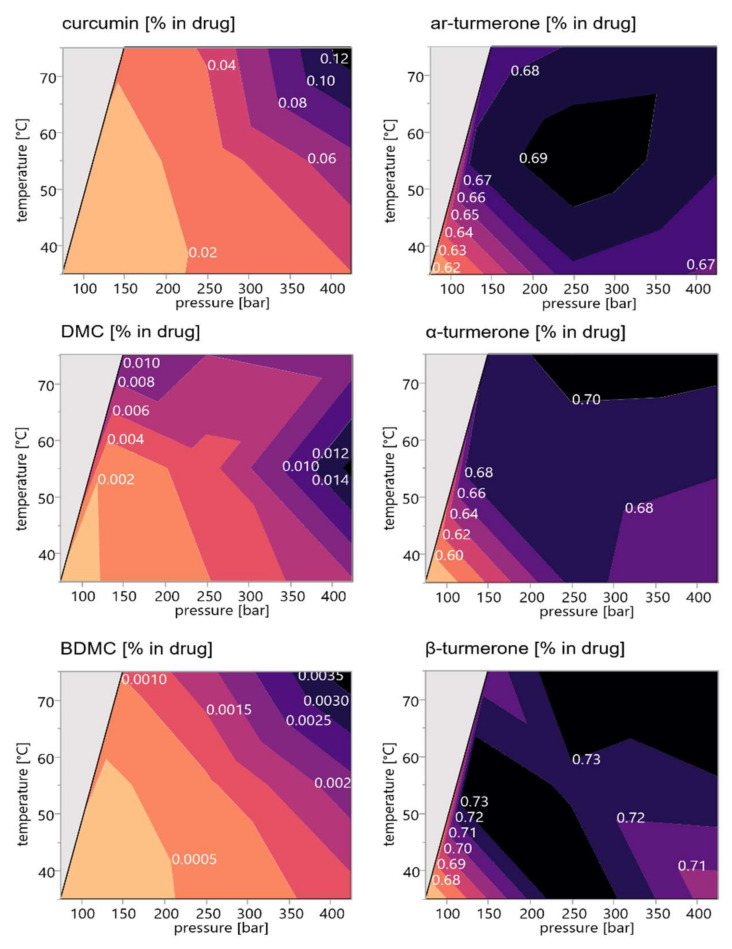
Response contour plots illustrating the recovery rates of all target compounds (curcumin, demethoxycurcumin (DMC), bisdemethoxycurcumin (BDMC), ar-turmerone, α-turmerone, and β-turmerone) depending on pressure and temperature; the areas marked in grey represent combinations of pressure and temperature settings, which were not investigated; recovery rates are related to the drug material in % (*m*/*m*).

**Figure 5:**
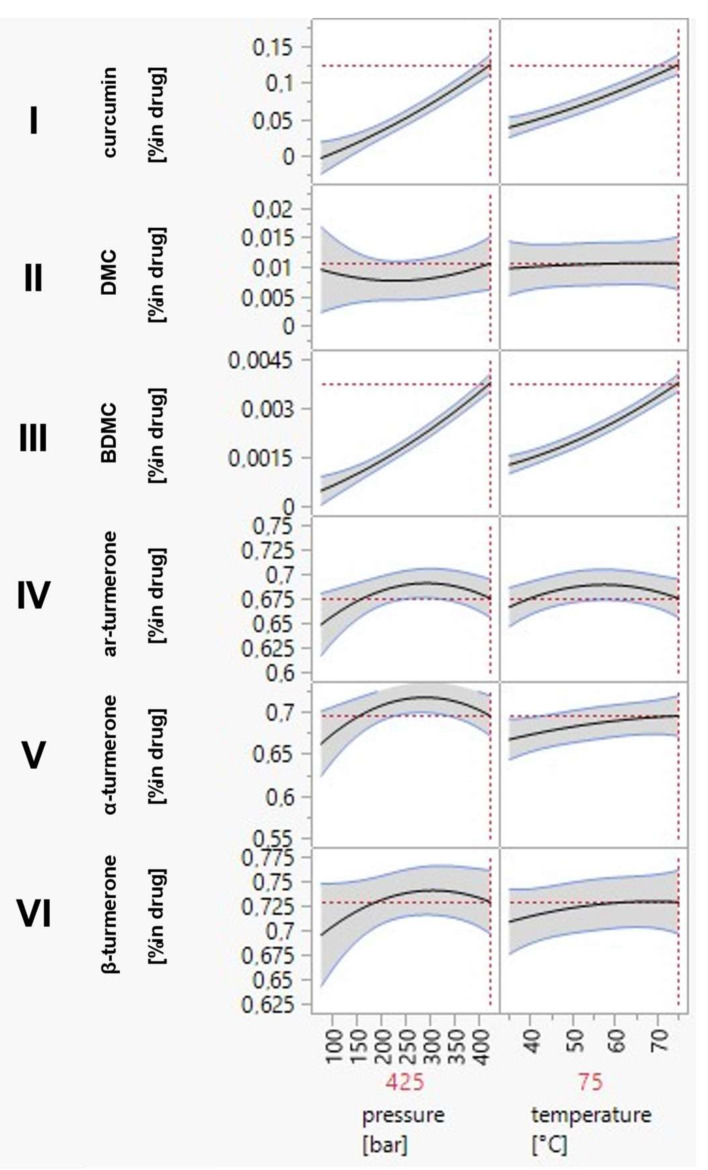
Prediction profiler diagrams with confidence intervals of a significance level of 0.05 show the separate influence of pressure and temperature on the recovery of all target compounds ((curcumin (**I**), demethoxycurcumin (**II**) (DMC), bisdemethoxycurcumin (**III**) (BDMC), ar-turmerone (**IV**), α-turmerone (**V**), β-turmerone (**VI**)). The optimum extraction conditions for maximal compound recovery are marked with the red dotted lines: 425 bar and 75 °C.

Figure 4 shows the response contour plots, which were calculated from the quantitative HPLC-DAD data of the marker substances of the scCO_2_ extracts. Both temperature and pressure have a clear impact on the recovery rates of the six main compounds. The curcumin and BDMC contents of the extracts were found to increase with increasing pressure and temperature, while the DMC content reached a maximum at maximum pressure and intermediate temperature settings. Curcumin extraction thereby ranged between <0.02% for low-to-intermediate pressure and temperature settings (75–200 bar and 35–60 °C) and >0.12% for maximum pressure and temperature settings (425 bar and 75 °C). For BDMC the extraction varied between <0.0005% (settings below 200 bar and 55 °C) and >0.0035% (settings above 400 bar and 70 °C), while DMC content ranged between <0.002% (settings below 125 bar and 50 °C) and >0.014% (settings of above 400 bar and 50–60 °C). The prediction profiler diagrams show the individual influence of temperature and pressure on extraction rates (Figure 4). A closer examination of the slopes and shapes of the curves in these diagrams reveals a much greater effect of temperature and/or pressure change on curcumin and BDMC recovery than on the yields of DMC and of the turmerones. Due to the steeper slope upon changing the pressure, the influence of this latter parameter can be considered most significant (Figure 5I–III). To optimize curcuminoid extraction would thus require maximum pressure conditions of 425 bar and intermediate to high temperature settings of 55–75 °C.

Turmerones generally have a better solubility in scCO_2_ due to their rather non-polar sesquiterpene structure. Nevertheless, they are considered to form part of the less-volatile essential oils [37,38]. They yielded maximal recoveries of <0.68–0.73% within a broad range of pressure and temperature settings (above 200 bar and 50 °C)), with medium-to-high pressure and temperature being advantageous, as shown in Figure 4. The steeper slope of the pressure dependent prediction profiler graphs for the turmerones (Figure 5IV–VI) indicates that pressure changes have a greater impact on their extraction rate than temperature changes. Furthermore, an extraction process aiming at maximal turmerone recovery would be performed best at medium pressure and temperature settings (250 bar/55 °C). This is consistent with previous publications [39,40].

The extraction behavior of turmerones and curcuminoids can be explained by their enhanced solubility in scCO_2_ with increasing density, which is clearly visible even at small pressure changes above the critical point (31.0 °C; 73.8 bar) [23,24,25]. Furthermore, scCO_2_ is described as a nondipolar, but it is also a quadrupolar solvent and a Lewis acid. This allows scCO_2_ to dissolve turmerones as well as the more polar curcuminoids [28].

Maximizing compound recovery for both curcuminoids and turmerones reveals optimum extraction conditions at 425 bar and 75 °C based on the DoE. If it would have been technically feasible to extend the design space to higher pressure and temperature conditions, it could be expected that the maximum would be found at more drastic conditions.

### 3.3. Comparison of scCO_2_ Extraction and Conventional Solvent Extraction

**Figure 6:**
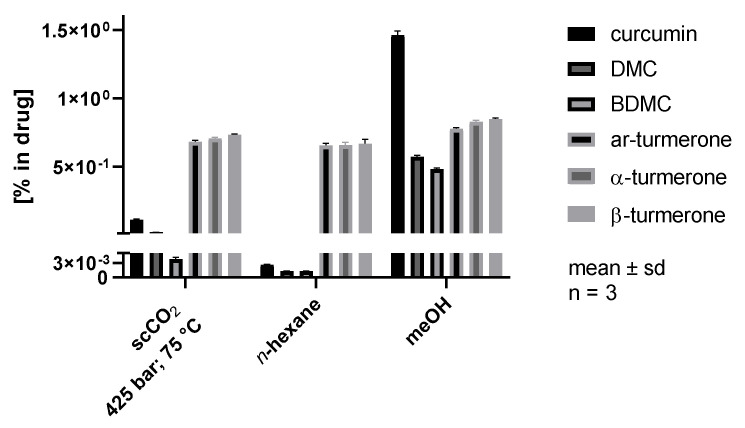
Comparison of a scCO_2_ extract characterized by maximum curcumin and ar-turmerone yields with *n*-hexane and methanol extracts.

Comparing the optimized scCO_2_ extract to *n*-hexane and methanol extracts reveals similar results for all three solvents when assessing turmerone recovery rates, yielding 0.66–0.86% (Figure 6). In contrast, curcuminoid extraction behavior differed significantly, with methanol yielding by far the highest curcuminoid concentrations of approx. 1.5% for curcumin, 0.57% for DMC, and 0.48% for BDMC, and *n*-hexane yielding the lowest amounts of <0.0026% for curcumin, 0.0013% for DMC, and 0.0013% for BDMC. The scCO_2_ extract showed intermediate curcuminoid concentrations of approx. 0.11% for curcumin, 0.02% for DMC, and 0.004% for BDMC, which is in line with expectations considering its polarity as a quadrupolar solvent and a Lewis acid [28]. This allows scCO_2_ to interact with the hydroxy and ether groups of the curcuminoids, which goes along with a solubility of these compounds ranging between the nonpolar *n*-hexane and the highly polar methanol. Without the use of potentially toxic solvents, both turmerones and curcuminoids can be extracted with scCO_2_. Thereby, the composition pattern can be varied using pressure and temperature settings.

### 3.4. Water Content Analysis

**Figure 7:**
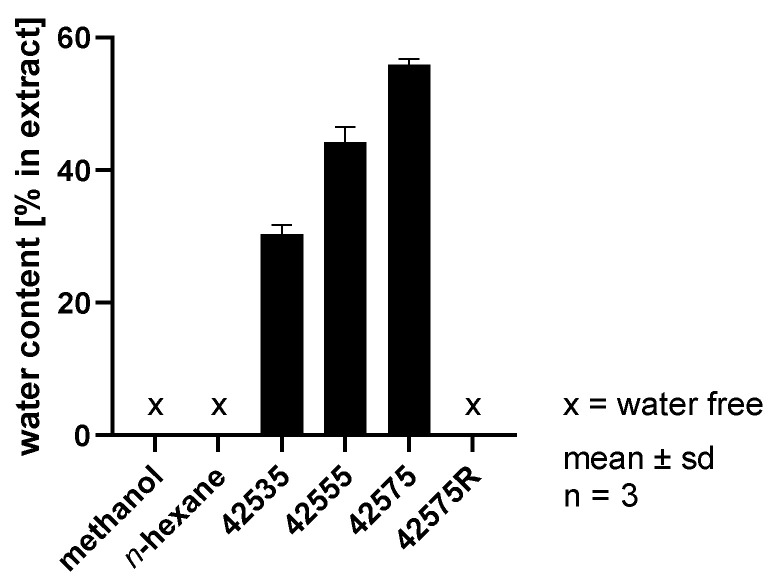
Water content in conventional solvent extracts with the solvents methanol and *n*-hexane and in scCO_2_ extracts produced at 425 bar and 35 °C (42535), 55 °C (42555), 75 °C (42575), and 75 °C dissolved in methanol with subsequent removal of solvent (42575R); determined using Karl Fischer semi-micro water determination.

It has been described in the literature that residual water in the drug is co-extracted during the scCO_2_ extraction process especially at higher temperatures [37]. To this end, the water content in the scCO_2_ extracts that were withdrawn directly from the separator according to Table 2 was determined (Figure 7). Expectedly, the water content for the three scCO_2_ extracts 42535, 42555, and 42575 increases with increasing temperature from approx. 30% for 42535 to 56% for 42575. The high amount of co-extracted water again underlines the role of scCO_2_ as a quadrupolar solvent. In contrast, the conventional solvent extracts as well as the scCO_2_ extract, that was taken from the plant as a methanolic solution with subsequent removal of the solvent, were considered to be water-free due to the solvent removal during production.

### 3.5. Stability Study with scCO_2_ Extracts

**Figure 8:**
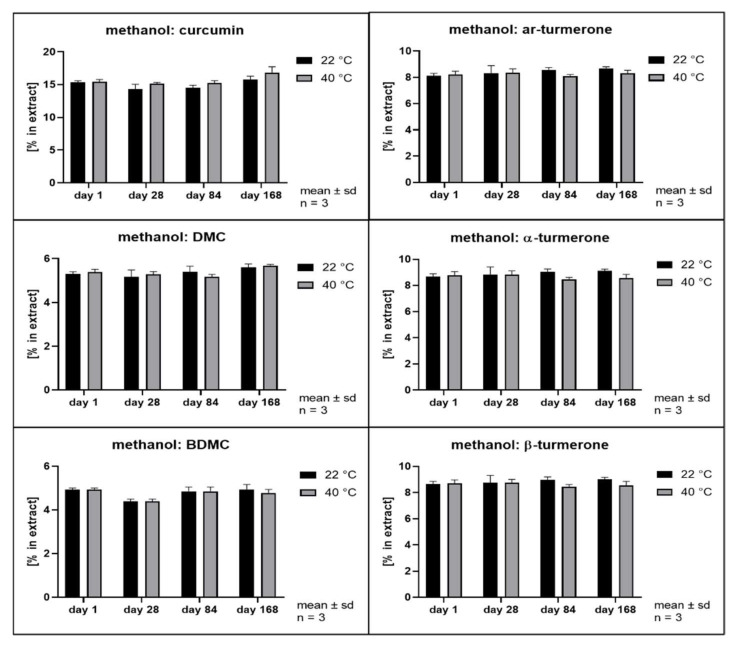
Contents of individual curcuminoids and turmerones in **methanolic solvent extracts** throughout storage.

**Figure 9:**
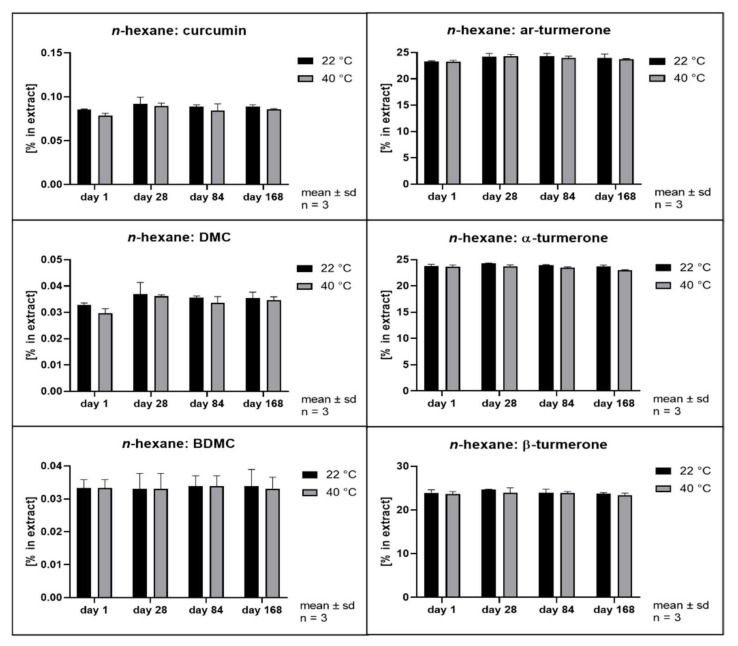
Contents of individual curcuminoids and turmerones in ***n*-hexane solvent extracts** throughout storage.

**Figure 10:**
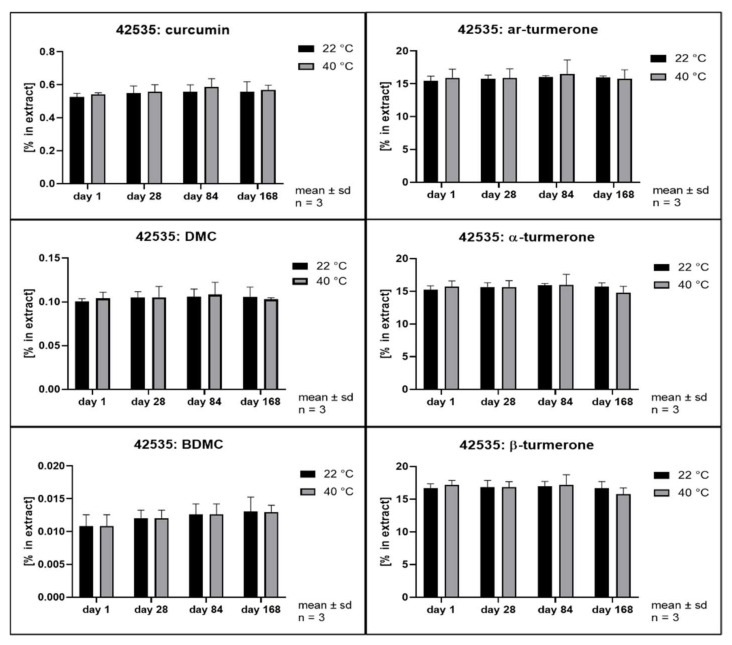
Contents of individual curcuminoids and turmerones in **scCO_2_ extracts** throughout storage, **extraction at 425 bar and 35 °C**.

**Figure 11:**
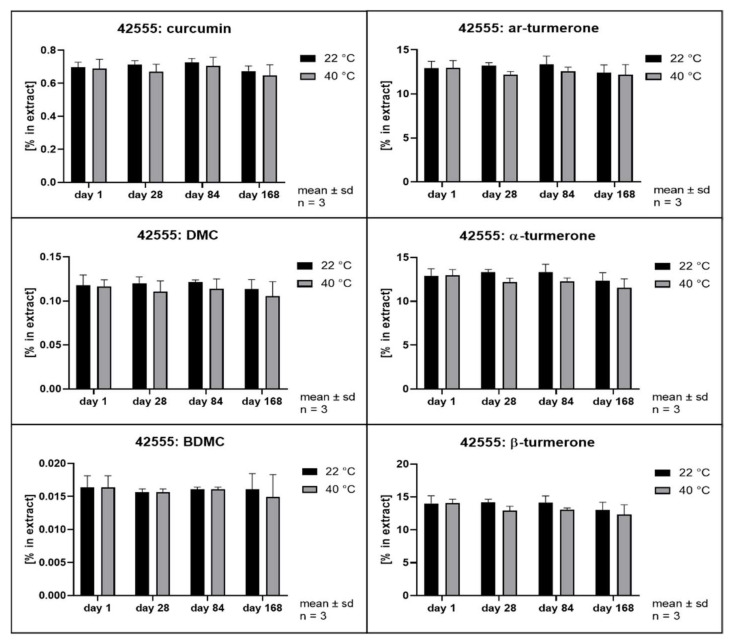
Contents of individual curcuminoids and turmerones in **scCO_2_ extracts** throughout storage, **extraction at 425 bar and 55 °C**.

**Figure 12:**
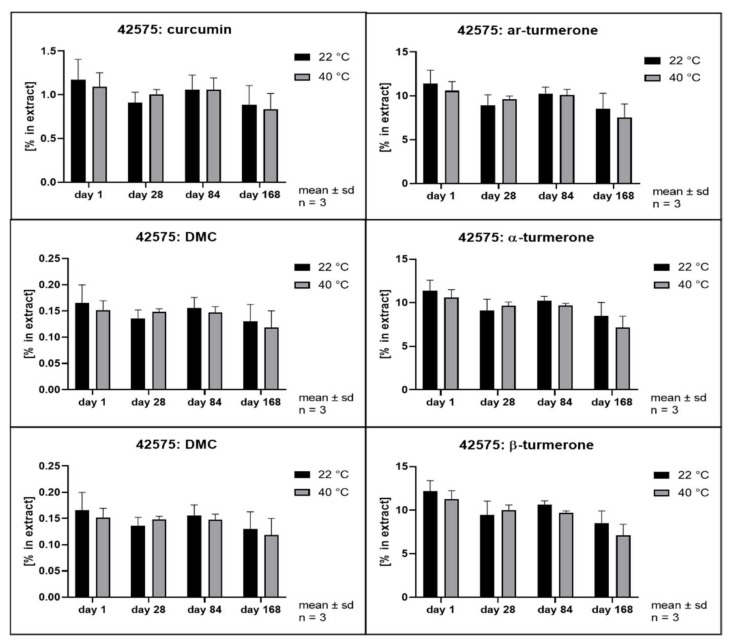
Contents of individual curcuminoids and turmerones in **scCO_2_ extracts** throughout storage, **extraction at 425 bar and 75 °C**.

**Figure 13:**
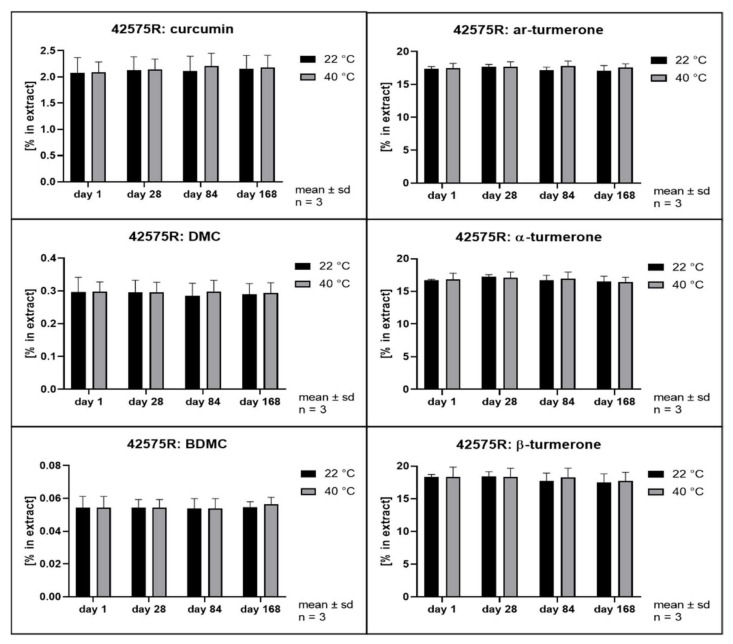
Contents of individual curcuminoids and turmerones in scCO_2_ extracts throughout storage, extraction at 425 bar and 75 °C, extract recovery via methanolic solution.

The hydrolytic degradation of curcumin has been described to follow second order kinetics and to be highly dependent on the pH value of the aqueous phase [3]. Its antioxidant activity has been explained as a degradation reaction driven by its phenolic groups [41]. In order to evaluate whether different solvents and extraction conditions upon scCO_2_ extraction affect the compound stability, samples were stored at 22 and 40 °C in the dark. Remarkably, all extracts separated into at least two phases after storage of only 28 days. The hexane extract and the scCO_2_ extracts separated into a clear oily phase on top of a cloudy semisolid phase. In addition, the scCO_2_ extracts that were directly recovered from the separator (42535, 42555, and 42575) developed a third phase consisting of small water drops at the bottom of the storage containers. The methanol extracts showed separation into a clear fluid oily phase at the bottom of the containers layered by a second phase which became harder and coarser with time. In the literature, the growth of curcumin crystals in aqueous solutions has already been described when the solubility limit is exceeded [42]. Likewise, methanol extracts showed the formation of coarse crystals. All other extracts remained either liquid or semisolid with visibly small crystals only. Thus, all extracts were thoroughly homogenized prior to sample analysis. Figure 8, Figure 9, Figure 10, Figure 11, Figure 12 and Figure 13 show the concentration profiles of the main compounds during six months of storage.

Both the methanol extract (Figure 8, methanol) and the *n*-hexane extract (Figure 9 *n*-hexane) revealed almost no changes in the concentration of the marker substances throughout storage. Figure 10, Figure 11 and Figure 12 show the contents of individual constituents of the scCO_2_ extracts that were produced under maximum pressure conditions (425 bar) and at different temperatures (35 °C, 42535; 55 °C, 42555; 75 °C, 42575). All these extracts were directly recovered from the separator without the addition of any further solvents. The extract produced at 35 °C did not show significant degradation of its constituents over the period considered. Degradation tendencies are evident in the extracts, which were prepared at higher temperatures. For the extract prepared at 55 °C there were slight degradation tendencies of approx. 10% for α- and β- turmerone and 5% for the curcuminoids and ar-turmerone which was observed after 168 days. The extract produced at 75 °C showed a statistically significant decrease of about 30% in content for all three turmerones after 168 days and a decrease of about 30% for the curcuminoids after 168 days, although that was not statistically significant. However, no degradation products were detected using HPLC. On the contrary, an extract that was also prepared at 425 bar and 75 °C but was dissolved in methanol for the removal from the separator showed no degradation during storage at all (Figure 13, 42575R).

Obviously, dissolving the extract in methanol and subsequent removal of the organic solvent improved the storage stability of the extract (42575 vs. 42575R). This follows an increase in the concentrations of the major components and a decrease in the water content (Figure 7). This can be explained by the simultaneous removal of water when methanol was removed, which in turn increased the content of the marker compounds in the remaining extract. Obviously, a higher water content in the extracts is responsible for the faster degradation seen in extracts that have been prepared at higher temperatures. Due to the presence of CO_2_, the pH value of the aqueous phase of the extracts was found to be slightly acidic [37]. Thus, a very poor solubility of the curcuminoids in this water phase can be assumed [3]. It has been described that the hydrolytic degradation of curcumin follows a second order kinetics and that the half-life at a pH between 3 and 6 ranges from 146 to 175 days [3]. This is almost in line with the results demonstrated for the curcuminoids of extract 42575 (Figure 12). The poor water solubility of curcuminoids and the lower water content of extracts recovered at lower temperatures might be an explanation for the low degradation rates of the curcuminoids in extracts 42535 and 42555. The relatively slow acid-catalyzed hydrolytic degradation of curcumin is further delayed by its pronounced solubility in the lipid phase, by its crystallization during separation from scCO_2_ following extraction, and by recrystallization during storage [42,43]. This further explains the more pronounced degradation of curcumin in the scCO_2_ extract with the highest water content (42575). Curcumin is known to be strongly susceptible to autoxidative degradation at neutral and basic pH [41,44,45] in aqueous surrounding, whereas ar-turmerone has been described to be degraded upon oxidative, photolytic, and thermal triggering [46]. This suggests that turmerone as well as curcuminoid degradation may be triggered by autooxidation in the presence of co-extracted water. In contrast, specific degradation routes have not been described in the literature for the turmerones. However, their highly lipophilic character alongside very poor solubility in water may be the reason for their very slow degradation even in the extract characterized by the highest water content (42575).

Interestingly, storage temperature (22 or 40 °C) only slightly and insignificantly affected the stability of the extracts as can be deduced from Figure 8 to Figure 13. This was unexpected as according to van’t Hoff’s rule a significantly higher reaction rate should result at higher temperatures [47]. However, our results are in line with the behavior of curcumin in acidic oil in water emulsions and solutions as published by Kharat et al. [42]. A possible explanation for this deviation from van’t Hoff’s rule might be the overlap of several effects including the very poor water solubility [48,49] and changes in distribution equilibrium and recrystallization due to distinct polymorphism of curcumin [43]. This results in a very complex scenario, which is typical of multicomponent mixtures but prevents the identification of monocausal explanations.

## 4. Conclusions

ScCO_2_ appears to be an ideal alternative for the extraction of essential oils from turmeric as illustrated for the turmerones. Even at relatively low temperatures the absolute amount of extracted turmerones in scCO_2_ extracts reaches its maximum and which is comparable to methanol extracts. The recovery rates of the curcuminoids, which are much more polar than the turmerones, can be enhanced by selecting optimal extraction conditions although their concentration remained about 15 times below that of a comparable methanol extract. Consequently, scCO_2_ extraction of turmeric is not directly comparable to conventional solvent extraction neither with methanol as a polar solvent nor with hexane as a non-polar solvent with regard to the compound profiles of the corresponding extracts.

Storage trials revealed the superior stability of all extracts compared to the scCO_2_ extract produced at 75 °C and 425 bar directly recovered from the extraction unit. Obviously, the co-extracted water negatively affects the extract stability. As the co-extracted amount of water increases with increasing extraction temperature and pressure, optimum scCO_2_ extraction conditions come with reduced storage stability. As a consequence of the reduced storage stability in the presence of water, removal of moisture must be considered before storing the scCO_2_ extracts. It is worthwhile to mention that pre-drying of the powdered turmeric is not recommended as it would affect the outcome of the scCO_2_ extraction process.

## Figures and Tables

**Table 1:** Experimental conditions according to a full factorial design in the correct experimental order.

Experiment	Pressure (bar)	Temperature (°C)
1	75	35
2	250	35
3	425	35
4	250	55
5	125	55
6	425	55
7	425	75
8	250	75
9	150	75
10	125	55
11	425	35
12	425	75
13	250	35
14	250	75
15	425	55
16	75	35
17	150	75
18	250	55
19	425	35
20	250	55
21	250	35
22	250	75
23	75	35
24	150	75
25	425	75
26	425	55
27	125	55
28	250	55
29	250	55
30	250	55
31	250	55
32	250	55
33	250	55

**Table 2:** Characteristics of the extracts monitored in the stability study.

Extract	Extraction Parameters	
methanol	methanol	solvent extract
*n*-hexane	*n*-hexane	solvent extract
42535	scCO_2_; 425 bar; 35 °C	direct removal
42555	scCO_2_; 425 bar; 55 °C	direct removal
42575	scCO_2_; 425 bar; 75 °C	direct removal
42575R	scCO_2_; 425 bar; 75 °C	removed as methanolic solution

**Table 3:** Retention times (R_t_), UV- and mass spectrometric data of active substances of turmeric. Only the most intense *m*/*z* ratios of fragment ions are displayed (^P^ precursor ion).

Peak No.	R_t_ (min)	Peak Assignment	UV λ_max_ (nm)	MS^n^ Data (*m*/*z*)			References
				MS ^1, P^	MS ^2, P^	MS ^3^	
1	24.5	bisdemethoxycurcumin (BDMC)	425	309	225	147	[30,31,32]
2	24.9	demethoxycurcumin (DMC)	425	339	245	175	[30,31,32]
3	25.3 Ref.: 25.3	curcumin	425	369	245	175	reference standard [30,31,32,33]
4	32.3 Ref.: 32.4	ar-turmerone	240	217	119	92	reference standard [34]
5	34.5 Ref.: 34.6	α-turmerone	236	219	121	93	reference standard [34,35,36]
6	34.7	β-turmerone	242	219	201	121	[34,35,36]

## Data Availability

Not applicable.
